# Supplementary material for: The mutational signatures of poor treatment outcomes on the drug-susceptible Mycobacterium tuberculosis genome
Source: eLife. 2023 May 3;12:e84815. doi: 10.7554/eLife.84815 (PMC10188106; doi:10.7554/eLife.84815)
Supplement: Figure 2—source data 1. [file elife-84815-fig2-data1.docx]

**Figure 2-source data 1. GWAS identified fixed SNPs.**

| **Position** | **Gene** | **SNP** | **GWAS** | **Proportion** | |
| --- | --- | --- | --- | --- | --- |
|  |  |  | **P value** | **Good** | **Poor** |
| 56142 | *Rv0051* | Q149H | 1.4×10^-7^ | 0.2% (7/3105) | 3.3% (3/91) |
| 121141 | *ctpB* | E345K | 1.7×10^-8^ | 1.2% (37/3105) | 5.5% (5/91) |
| 312445 | *Rv0260c* | T72I | 5.6×10^-8^ | 1.8% (56/3105) | 7.7% (7/91) |
| 744078 | *Rv0648* | P454S | 4.5×10^-8^ | 3.8% (119/3105) | 11.0% (10/91) |
| 1389358 | *Rv1248c* | *1232S | 1.5×10^-7^ | 3.8% (119/3105) | 12.1% (11/91) |
| 1974200 | *Rv1747* | T191A | 2.1×10^-7^ | 4.8% (148/3105) | 13.2% (12/91) |
| 2253677 | *otsB1* | G559D | 7.3×10^-10^ | 0.6% (18/3105) | 5.5% (5/91) |
| 2318502 | *cobN* | A751V | 6.4×10^-9^ | 4.7% (146/3105) | 13.2% (12/91) |
| 2427541 | *Rv2164c* | D233G | 4.0×10^-8^ | 4.2% (131/3105) | 12.1% (11/91) |
| 2482128 | *dlaT* | V55A | 2.1×10^-8^ | 4.3% (132/3105) | 13.2% (12/91) |
| 3537023 | *Rv3168* | E308* | 1.8×10^-7^ | 0.7% (22/3105) | 4.4% (4/91) |
| 3727924 | *metA* | G146D | 2.2×10^-8^ | 4.6% (144/3105) | 13.2% (12/91) |
| 3727933 | *metA* | E149G | 8.8×10^-8^ | 4.8% (148/3105) | 13.2% (12/91) |
| 4291685 | *papA1* | I497T | 1.0×10^-7^ | 0.1% (4/3105) | 2.2% (2/91) |
| **Total** |  |  |  | 7.5% (232/3105) | 24.2% (22/91) |

*Asterisks indicate stop codons.
